# Iron Levels and Markers of Inflammation in a Population of Adults with Severe Obesity, a Cross-Sectional Study

**DOI:** 10.3390/nu15214702

**Published:** 2023-11-06

**Authors:** Daniela Laudisio, Giulia de Alteriis, Claudia Vetrani, Sara Aprano, Gabriella Pugliese, Francesca Zumbolo, Annamaria Colao, Silvia Savastano

**Affiliations:** 1 Department of Public Health, University of Naples Federico II, Via Sergio Pansini 5, 80131 Naples, Italy; 2 Centro Italiano per la Cura e il Benessere del Paziente con Obesità (C.I.B.O), Unità di Endocrinologia, Diabetologia e Andrologia, Dipartimento di Medicina Clinica e Chirurgia, Università degli Studi di Napoli Federico II, Via Sergio Pansini 5, 80131 Naples, Italy; claudia.vetrani@gmail.com (C.V.); apranosara90@gmail.com (S.A.); acolao@unina.it (A.C.);; 3 Unità di Endocrinologia, Diabetologia e Andrologia, Dipartimento di Medicina Clinica e Chirurgia, Università degli Studi di Napoli Federico II, Via Sergio Pansini 5, 80131 Naples, Italy; dealteriisgiulia@gmail.com (G.d.A.); robiniapugliese@gmail.com (G.P.); francesca.zumbolo@gmail.com (F.Z.); 4 Dipartimento di Scienze Umanistiche, Università Telematica Pegaso, Via Porzio, Centro Isola F2, 80143 Napoli, Italy; 5 Cattedra Unesco “Educazione Alla Salute e Allo Sviluppo Sostenibile”, Università degli Studi di Napoli Federico II, Via Sergio Pansini 5, 80131 Naples, Italy

**Keywords:** serum iron levels, obesity, visceral obesity, waist circumference, c-reactive protein, low-grade chronic inflammation

## Abstract

Low-grade chronic inflammation linked to obesity can lead to alterations in biomarkers of iron status. The aim of this study was to investigate the primary determinant of serum iron levels among anthropometric measurements, body fat, and serum biomarkers of low-grade chronic inflammation in a group of adult individuals with severe obesity. We enrolled 114 individuals (84 females; 30 males) aged 40.96 ± 12.54 years. Weight and body mass index (BMI) were 121.20 ± 22.33 kg and 44.94 ± 7.29 kg/m^2^, respectively. Some 30% of individuals had class-II obesity (BMI ≥ 35 ≤ 39.9 kg/m^2^) and 70% had class-III obesity (BMI ≥ 40 kg/m^2^). A weak, albeit significant, inverse correlation was found between serum iron levels and c-reactive protein (CRP) (r = −0.259, *p* = 0.008), fibrinogen (r = −0.261, *p* = 0.006), BMI (r = −0.186, *p* = 0.04), waist circumference (WC) (r = −0.265, *p* = 0.004), and fat mass % (r = −0.285, *p* = 0.003). With multiple linear regression analysis including CRP, fibrinogen, BMI, WC, and fat mass % as independent variables and serum iron levels as dependent variable, WC was entered in the first step (*p* = 0.001), which was followed by fat mass % (*p* = 0.047) and CRP (*p* = 0.047). Grouping the individuals according to the interquartile range of BMI, WC, and fat mass % (Q1–Q4), the lowest serum iron levels were found in Q4 groups of WC and fat mass % (*p* = 0.02), while no significant differences were found between groups in BMI quartiles. In conclusion, in our study, population serum iron levels were inversely associated with BMI, visceral obesity, fat mass %, CRP, and fibrinogen, but WC was the major negative predictor of serum iron level. These results supported the fact that visceral distribution of body fat, more than obesity per se, was associated with low serum iron levels in adult individuals with severe obesity.

## 1. Introduction

Iron-deficiency anaemia and iron deficiency associated with obesity are a worldwide public health problem [1]. Several studies have suggested that obesity is associated with low serum iron levels and iron deficiency [2,3,4]. In particular, a meta-analysis showed that, compared with non-overweight participants, overweight/obese participants had lower serum iron concentrations and a significantly increased risk of iron deficiency [3]. However, the method used to diagnose iron deficiency can have a critical effect as the authors found a significant correlation between iron deficiency and obesity in studies without a ferritin-based diagnosis, but not in studies that used a ferritin-based diagnosis [3]. Indeed, as also reported by Khan A et al. [5], ferritin could serve as a marker of inflammation rather than iron deficiency in individuals who were overweight/obese [5]. In this context, Flancbaum et al. [6] reported preoperative deficiencies of 43.9% for iron, but only 8.4% for ferritin among patients scheduled for bariatric surgery [6]. Therefore, in patients with chronic systemic inflammatory conditions, such as those affected with obesity, mainly visceral obesity, elevated serum ferritin might not correlate with body iron status [7]. It is well known that body mass index (BMI) is a proxy measure for obesity because it does not directly measure body fat, and nor does it provide information on the ectopic location of fat deposition, which has been found to be more closely related to low-grade chronic inflammation [8]. Considering that waist circumference (WC) is the best anthropometric indicator of visceral fat, the evaluation the iron status only based on degree of obesity could induce possible pitfalls in the diagnosis of iron deficiency in individuals with obesity. Recently, Kerkadi et al. [4] investigated the link between abdominal obesity and iron status among adults in Quatar [4]. The results showed that in women, serum iron levels and transferring saturation decreased significantly along with the increase in waist circumference (WC), while in both genders C-reactive protein (CRP) levels increased with the increase in WC and BMI [4]. The present study aimed to investigate the primary determinant of iron serum levels among anthropometric measurements, body fat, and serum biomarkers of low-grade chronic inflammation in a group of adult individuals with severe obesity.

## 2. Materials and Methods

### 2.1. Design and Setting

This was a cross-sectional observational study performed at the Endocrinology Unit and General Surgery Unit of the Federico II University (Naples, Italy). In this study, individual candidates for bariatric surgery were consecutively enrolled from April 2022 to April 2023. All individuals underwent a nutritional and metabolic evaluation after providing written informed consent. The study protocol, which has been approved by the Ethical Committee of the University of Naples Federico II (n. 173/16), was executed according to the Code of Ethics of the World Medical Association (Declaration of Helsinki) for experiments involving humans and patients, who were extensively informed before signing the consent.

### 2.2. Population Study

All the individuals underwent a multidisciplinary preoperative evaluation, including demographic characteristics (age, gender), medical history and current medications, physical examination, electrocardiogram, urine analysis, and screening blood tests (blood urea nitrogen, creatinine, uric acid, albumin, aspartate aminotransferase and alanine aminotransferase, glucose, insulin, glycated haemoglobin (HbA1c), total cholesterol, low-density lipoprotein (LDL) cholesterol, triglycerides (TG), albumin, iron, and haemoglobin). Eligible participants for the study were adult individuals aged 18–65 years with BMI ≥ 35 kg/m^2^. Exclusion criteria were altered liver, cardiopulmonary, and kidney functions and endocrine diseases including hypothyroidism, Cushing’s syndrome, and hypogonadism. Other criteria for exclusion were drug or alcohol abuse and iron supplements in the 3 months prior to the enrolment. Individuals following a particular dietary regimen for any reason were also excluded from this study.

### 2.3. Anthropometric Measurements

Anthropomorphic assessment (height, weight) was performed, and BMI was calculated. The measurements were carried out in the morning between 8:30 a.m. and 12:00 p.m. after an overnight fast. The anthropometric measurements were taken following standard criteria from the same nutritionist according to the International Society for the Advancement of Kinanthropometry (ISAK 2006). BMI (weight (kg) divided by height squared (m^2^), kg/m^2^) was calculated after measuring weight and height. WC was measured to the closest 0.1 cm with a non-extensible tape. The measurements were performed with the individual standing, feet together, and arms hanging freely at the sides, with the individuals standing and breathing normally. WC was measured at the midpoint between the inferior costal margin and the upper iliac crest. Hip circumference (HC) was measured as the maximum circumference around the buttocks posteriorly and the symphysis pubis anteriorly, and measured to the nearest 0.5 cm; the waist-to-hip ratio (WHR) was calculated.

### 2.4. Bioelectrical Impedance Analysis

Bioelectrical impedance analysis has been used for assessed body composition (an 800 µA current at a single frequency of 50 kHz BIA 101 RJL, Akern Bioresearch, Florence, Italy). The analysis was performed according to the European Society of Parental and Enteral Nutrition (ESPEN) [9]. The same operator and the same device obtained bioelectrical impedance analysis determinations under strictly standardised conditions in order to avoid interobserver and interdevice variability. The bioelectrical impedance analysis was routinely checked with resistors and capacitors of known values. [10].

### 2.5. Assay Methods

Venous blood samples were taken from the antecubital vein between 8 and 10 a.m. after an overnight fast of at least 8 h, collected in vacutainer tubes containing EDTA, and stored at −80 °C until processed. All biochemical analyses included total cholesterol, TG, and were carried out with a Roche Modular Analytics System in the Central Biochemistry Laboratory of our Institution. LDL and high-density lipoprotein (HDL) cholesterol were quantified by a direct method (homogeneous enzymatic assay for the direct quantitative determination of LDL and HDL cholesterol). Fasting plasma glucose concentration was measured by the glucose oxidase method. HbA1c was measured with high-performance liquid chromatography (HPLC). For serum iron levels, an iron ferrozine complex method was used with sensitivity 5 µg/dL, and the serum iron normal range was 50–175 µg/dL. Serum levels of 25OHD were quantified by a direct competitive chemiluminescence immunoassay (CLIA) (Liaison, DiaSorin, Saluggia, Italy), with a specificity of 100% for 25OHD [11]. Serum hs-CRP levels were measured with Siemens Healthcare Diagnostics (Marburg, Germany) with a nephelometric assay with CardioPhase high sensitivity. The intra- and inter-assay coefficients of variation were <7%. The fasting insulin levels were determined by a solid-phase chemiluminescent enzyme immunoassay using commercially available kits (Immulite Diagnostic Products Co., Los Angeles, CA, USA). Homeostasis model assessment was calculated according to Matthews et al. [12]. A homeostasis model assessment value of > 2.5 was used as cut-off of IR. Haemoglobin and mean corpuscular volume were quantified using flow cytometry (CELL-DYN^®^ 4000; Abbott Laboratories, Abbott Park, IL, USA).

### 2.6. Statistical Analysis

The data distribution has been evaluated by a Kolmogorov–Smirnov test, and the abnormal data were normalised by a logarithm. Skewed variables were back-transformed for presentation in tables and figures. Results are shown as mean ± SD and categorical variables are shown as percentages. Differences between individuals with II- and III-grade obesity were assessed by a Student’s independent samples *t*-test. The correlations among serum iron levels and BMI, WC, CRP, fibrinogen, and fat mass % were analysed with the Pearson *r* correlation coefficient. Multivariate logistic regressions were performed to assess the associations between serum iron levels and WC. Differences between WC quartiles were analysed by one-way ANOVA with a post-hoc test for multiple comparisons. The level of significance was taken as *p* < 0.05. Data were analysed using SPSS Software (PASW Version 21.0; SPSS Inc., Chicago, IL, USA) and the MedCalc^®^ package (Version 12.3.0 1993–2012 MedCalc Software bvba—MedCalc Software, Mariakerke, Belgium). For the calculation of the sample size, we considered a 9% prevalence of severe obesity among adults [13]. Considering a type-I/-II error rate of alpha 0.05, beta 0.1 and a power size of 90%, the number of individuals to be enrolled was found to be 90. Considering a drop-out of 20%, the minimum number of cases required was 108. The calculation of the sample size was performed using clinical software (https://clincalc.com/stats/samplesize.aspx, accessed on 4 April 2023).

## 3. Results

The study cohort included 114 participants: 84 (73.7%) women and 30 (26.3%) men. Age, anthropometric characteristics, fat mass (kg), and fat mass percentage (%) of the study population are shown in Table 1. Some 34 individuals (29.8%) had class-II obesity (F/M = 22/12) and 80 (70.2%) had class-III obesity (F/M = 62/18). The metabolic characteristics are reported in Table 2.

Some 67 individuals (58.8%) showed 25(OH)D deficiency, and 25 (21.9%) and 11 (9.6%) presented folate and vitamin B12 deficits, respectively. Only 10 individuals (8.8%) showed anaemia and 23 (20.2%) showed low serum iron levels (Table 3).

The characteristics of the samples stratified by BMI categories are summarised in Table 4. There were significant differences between individuals with grade-II obesity and those with grade-III obesity. The mean value of serum iron levels was significantly lower in individuals with grade-III obesity than those with grade-II obesity (86.38 ± 37.71 vs. 72.01 ± 25.74, *p* = 0.02). The mean values of hs-CRP, fibrinogen, insulin, and homeostasis model assessment were significantly higher in individuals with grade-III obesity than grade-II obesity (0.76 ± 0.53 vs. 1.73 ± 1.75, *p* = 0.005), (338.44 ± 72.27 vs. 416.37 ± 104.48, *p* < 0.0001), (17.07 ± 8.03 vs. 23.28 ± 12.75, *p* = 0.015), and (3.89 ± 2.14 vs. 5.49 ± 2.93, *p* = 0.008), respectively. Furthermore, the individuals with grade-III obesity showed major nutritional deficiency in vitamin B12 (358.22 ± 130.33 vs. 335.56 ± 111.94), folic acid (5.20 ± 3.6 vs. 4.01 ± 2.21), and 25(OH)D (19.06 ± 9.75 vs. 16.27 ± 9.78), but the differences between the two groups were not significant.

### Correlation Study

**Figure 1:**
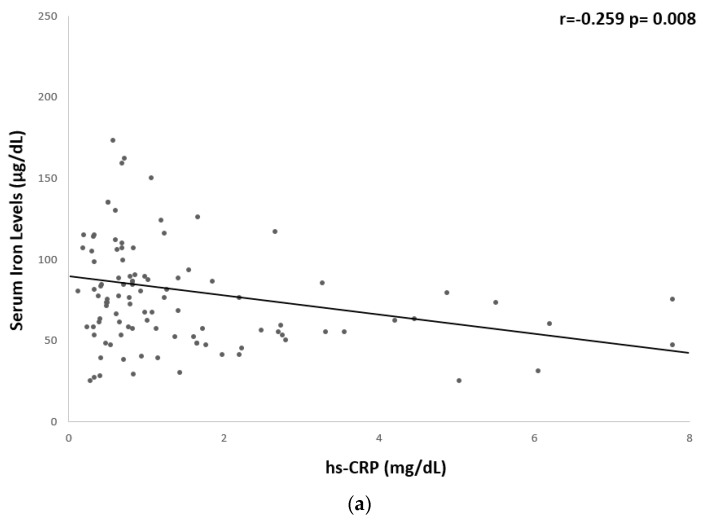

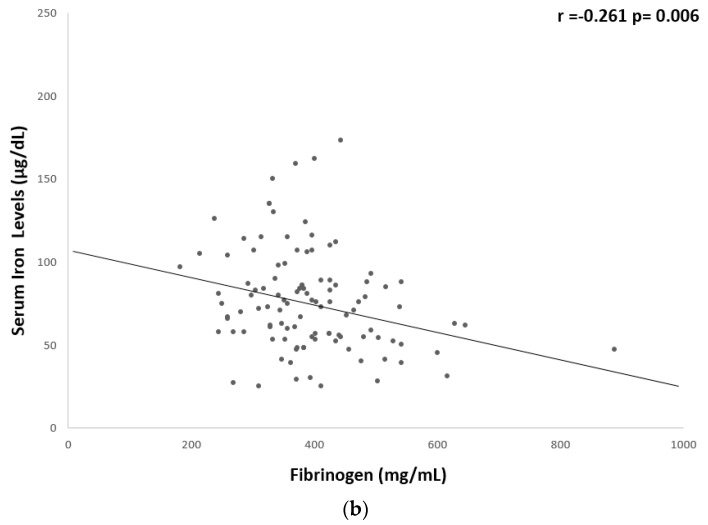
Univariate associations between serum iron levels and biological markers of low-grade chronic inflammation. Serum iron levels showed a significant but weak correlation with (**a**) hs-CRP (r = −0.259 *p* = 0.008) and (**b**) fibrinogen (r = −0.261 *p* = 0.006). CRP, c-reactive protein.

**Figure 2:**
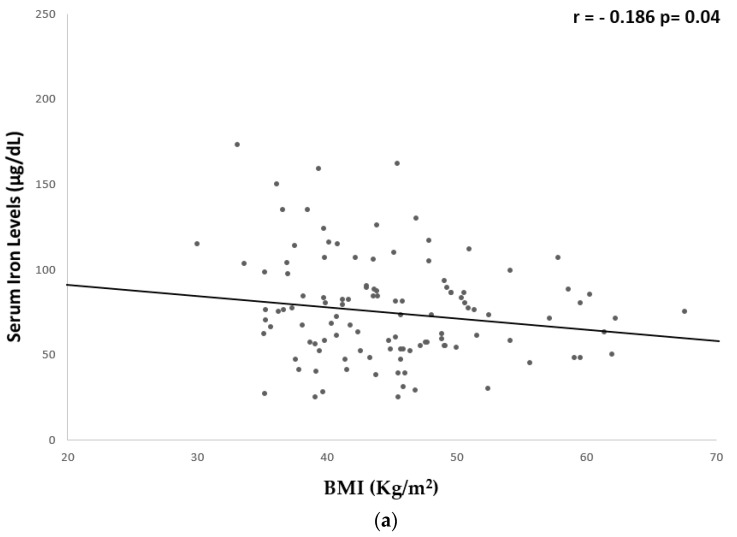

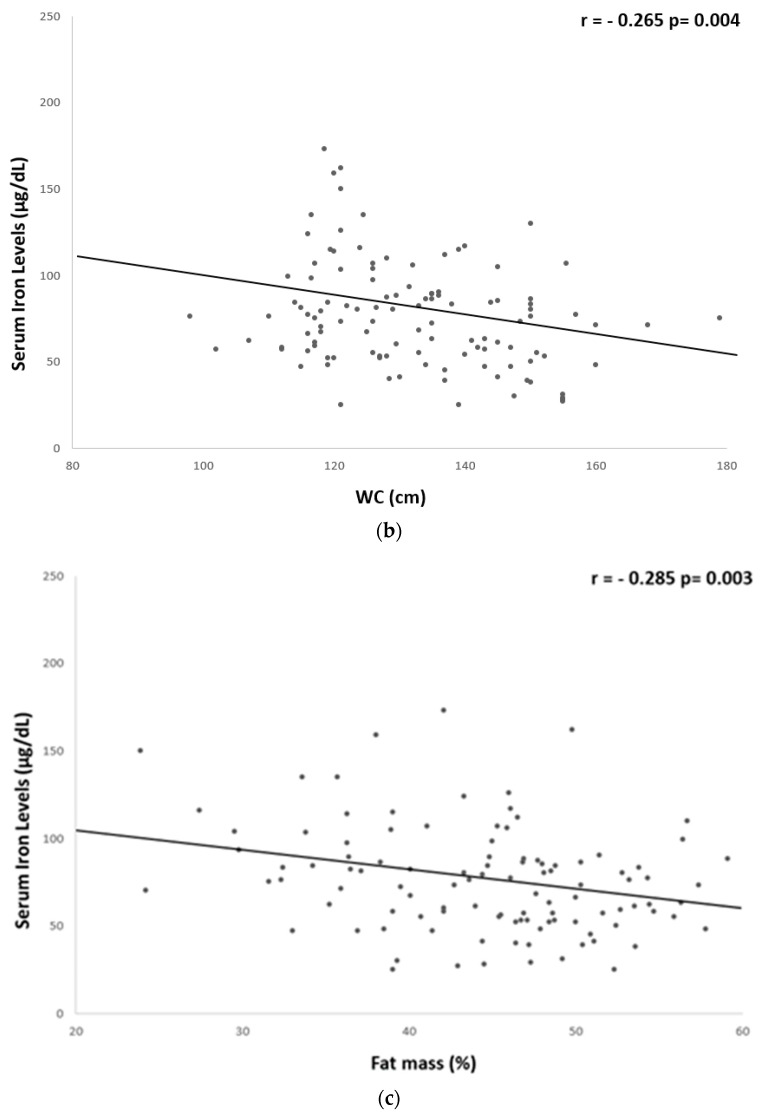
Univariate associations between serum iron levels and anthropometric parameters. Serum iron levels showed a significant but weak correlation with: (**a**) BMI (r = −0.186 *p* = 0.04); (**b**) WC (r = −0.265 *p* = 0.004); and (**c**) fat mass % (r = −0.285 *p* = 0.003). BMI, body mass index; WC, waist circumference.

A significant but weak inverse correlation was found between serum iron levels and hs-CRP (r = −0.259, *p* = 0.008), fibrinogen (r = −0.261, *p* = 0.006), BMI (r = −0.186, *p* = 0.04), WC (r = −0.265, *p* = 0.004), and fat mass % (r = −0.285, *p* = 0.003) (Table 5 and Figure 1 and Figure 2).

**Figure 3:**
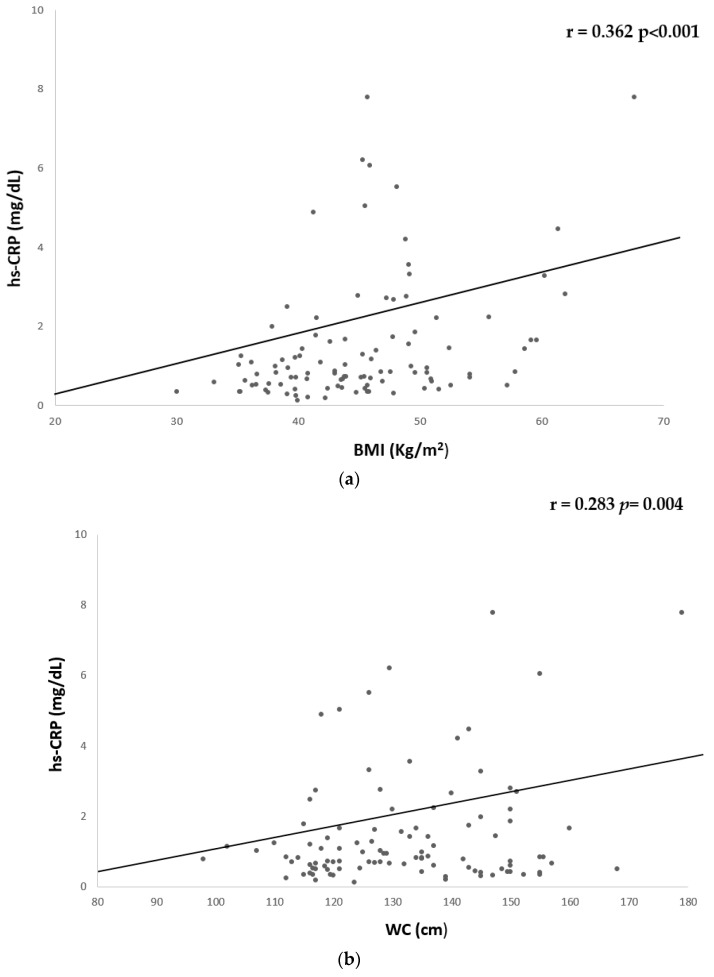
Univariate associations between hs-CPR and anthropometric parameters. hs-CRP showed a significant but weak correlation with (**a**) BMI (r = 0.362 *p* < 0.001) and (**b**) WC (r = −0.283 *p* = 0.004). CRP, c-reactive protein; BMI, body mass index; WC, waist circumference.

Moreover, a significant positive but weak correlation was found between hs-CRP and BMI (r = 0.362, *p* < 0.001) and WC (r = 0.217, *p* = 0.028) (Table 6 and Figure 3).

To compare the relative predictive power of hs-CRP levels, fibrinogen levels, BMI, WC, and fat mass % on serum iron levels, we performed multiple linear regression analysis using a model that included as independent variables hs-CRP, fibrinogen, BMI, WC, and fat mass % and serum iron levels as dependent variable. Using this model, WC was entered in the first step (*p* = 0.001), which was followed by fat mass % (*p* = 0.047) and hs-CRP (*p* = 0.047) (Table 7).

**Figure 4:**
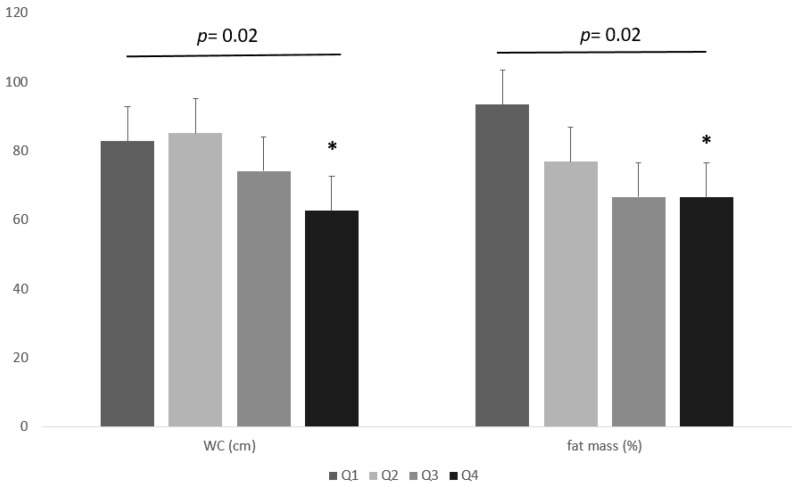
Differences in serum iron levels between the groups for WC and % fat mass quartiles. * One-way ANOVA with post hoc test for multiple comparisons.

In addition, individuals were grouped into four groups (Q1–Q4) according to the interquartile range of the WC, BMI, and fat mass %, as follows: lowest quartile ≤120 cm, second quartile = 120–130 cm, third quartile = 131–144 cm, and highest quartile ≥144 cm; lowest quartile ≤39.6 kg/m^2^, second quartile = 39.7–43.8 kg/m^2^, third quartile = 43.9–49.1 kg/m^2^, and highest quartile ≥ 49 kg/m^2^; lowest quartile ≤ 39.9%, second quartile = 40–46%, third quartile = 46.1–50%, and highest quartile ≥ 50.1%, respectively. Statistical differences in serum iron levels were noted between the groups for WC and % fat mass quartiles. No significant differences were found between groups in BMI quartiles. The lowest serum iron levels were found in the Q4 group for WC and fat mass % (Figure 4).

## 4. Discussion

In our study in adults with severe obesity, we evidenced an inverse correlation of serum iron levels with the anthropometric measurements, fat mass %, and serum markers of inflammation. Individuals with class-III obesity showed lower serum iron levels than those with class II. Moreover, low-grade chronic inflammation is higher in individuals with class-III obesity than in those with class-II obesity. However, as novel findings, we reported that the lowest serum iron levels were observed in individuals in the highest quartiles of WC and fat mass %, while no differences were evidenced along the quartiles of BMI. In addition, among anthropometric characteristics, fat mass %, and serum markers of inflammation, we found that the major determinants of serum iron levels remained the distribution of the body fat evaluated by WC.

The inverse association of iron levels with WC and biomarkers of low-grade chronic inflammation further supported the fact that low serum iron concentration associated with central obesity might be attributed to obesity-related inflammatory status. It is well known that visceral adipose tissue is more heavily infiltrated by macrophages producing interleukin (IL)-6 that is released into the portal circulation and stimulates hepatocytes to increase CRP synthesis [15,16]. In addition, interleukin (IL)-6 also modulates the concentration and biological activity of hepcidin, a peptide hormone mainly produced by the liver that reduces iron efflux from hepatocytes and duodenal iron absorption by reducing the stability of the ferroportin transporter [17,18,19]. In adults, a negative association was also evidenced between ferritin and indexes of distribution of adipose tissues in the body, including the visceral fat area, determined by computed tomography [20]. It is well known that ferritin is a key regulator of iron homeostasis that serves as an important clinical indicator of body iron status. Therefore, serum ferritin is useful biomarker for detecting iron deficiency in the absence of inflammatory conditions [21]. Among healthy patients, serum ferritin levels are directly proportional to iron status, and low serum ferritin is a highly specific and sensitive marker for diagnosing iron deficiency [7]. However, diagnosing iron deficiency might be more complex in cases of inflammatory conditions [22]. Indeed, as ferritin is an acute-phase reactive protein [23], its determination could lead to conflicting results in the diagnosis of iron deficiency in patients with obesity-related inflammatory status. In inflammatory states, reduced serum iron levels are associated with iron trapping within macrophages and liver cells, and reduced intestinal iron absorption [24,25]. Previous studies [18,26,27] reported an inverse association between serum iron levels and body fat distribution evaluated by dual-energy X-ray absorptiometry (DXA), the gold standard method for evaluating body composition [27]. However, due to mild radiation, DXA examinations should be limited to no more than two per year. In clinical practice, bioelectrical impedance analysis represents a more feasible method to assess body fat [28,29]. An inverse association of serum iron levels with fat mass % and abdominal–visceral fat mass evaluated by bioelectrical impedance analysis was reported in both sexes of preadolescents with obesity [30]. In line with these findings, we confirmed the inverse associations of serum iron levels with fat mass % and also WC in adults with severe obesity. In addition, as also above mentioned, we found that between fat mass % and WC, the latter was the main determinant of serum iron levels in the regression analysis.

There are some limitations to this study. The major limitations are the cross-sectional design of the study that does not allow any cause–effect conclusions and the lack of control group. In addition, the transferrin saturation was not assessed. Therefore, we cannot diagnose iron deficiency in this study. A third limitation might be the female preponderance (74%) in our study. This is, however, not unusual among individuals with severe obesity, as women are more prone to weight loss surgery [31]. Strengths of this study include a sufficiently adequate sample size; the determination of three different criteria to characterise obesity in our population sample, including BMI, WC, and fat mass %; and the evaluation of biomarkers of low-grade chronic inflammation. This allowed us to evidence that distribution of body fat was the primary determinant of low serum iron levels in adult individuals with severe obesity.

## 5. Conclusions

In conclusion, the present study aimed to provide further insights into the association of low serum levels in obesity, showing that, as previously reported in preadolescents with obesity, in adult individuals with severe obesity, serum iron levels were also lower in individuals presenting higher quartiles of WC and fat mass % compared to the corresponding lowest quartiles. In addition, our results highlighted that among the anthropometric measurements, fat mass %, and serum biomarkers of low-grade chronic inflammation, visceral adiposity evaluated by WC was the main predictor of serum iron levels. These results supported the fact that visceral distribution of body fat, more than obesity per se, is associated with low serum iron levels in adult individuals with severe obesity and suggested the usefulness of the evaluation of body fat distribution in the diagnosis of low serum iron levels in obesity. However, a focused study design on different population samples of individuals with obesity, also including hepcidine and other markers of iron status, are needed to confirm our findings.

## Figures and Tables

**Table 1:** Anthropometric characteristics of the study population.

Parameters	Mean ± SD or Number (%) n = 114
Males n. (%)	30 (26.3%)
Females n. (%)	84 (73.7%)
Age (years)	40.96 ± 12.54
BMI (Kg/m2)	44.94 ± 7.29
Grade II obesity n. (%)	34 (29.8%)
Grade III obesity n. (%)	80 (70.2%)
WC (cm)	132.20 ± 14.98
HP (cm)	138.36 ± 13.63
WHR	0.96 ± 0.09
Fat mass (%)	44.49 ± 7.60
Fat mass (kg)	53.38 ± 14.01
Free fat mass (%)	55.51 ± 7.60
Free fat mass (kg)	65.77 ± 12.55

Data are expressed as mean ± SD or n (%). A *p* value in bold type denotes a significant difference (*p* < 0.05); BMI, body mass index; WC, waist circumference; HP, hip circumference; WHR, waist-to-hip ratio.

**Table 2:** Metabolic characteristics of the study population.

Parameters	Mean ± SD n = 114
Glucose (mg/dL)	97.18 ± 23.77
Insulin (mIU/mL)	21.44 ± 11.86
HOMA-index	4.56 ± 3.04
HbA1c (%)	5.31 ± 1.53
Total cholesterol (mg/dL)	191.32 ± 37.20
LDL cholesterol (mg/dL)	133.17 ± 36.72
TG (mg/dL)	132.25 ± 67.66
Albumin (g/dL)	4.35 ± 0.30
Iron (μg/dL)	76.30 ± 30.37
Haemoglobin (g/dL)	13.65 ± 2.31
Vitamin B12 (pg/mL)	343.72 ± 118.52
Folic acid (ng/mL)	4.45 ± 2.83
25(OH)D (ng/mL) hs-CRP (mg/dL) Fibrinogen (mg/mL)	17.16 ± 9.81 1.47 ± 1.58 393.28 ± 102.19

Data are expressed as mean ± SD or n (%). A *p* value in bold type denotes a significant difference (*p* < 0.05); HbA1c, glycated haemoglobin; TG, triglycerides; HOMA, homeostatic model assessment; LDL, low-density lipoprotein cholesterol; HDL, high-density lipoprotein; 25(OH)D, 25-hydroxyvitamin D; CRP, c-reactive protein.

**Table 3:** Nutritional deficiency of the study population.

Parameters		Values
25(OH)D deficiency ^a^	n. (%)	67 (58.8%)
Folate deficiency ^b^	n. (%)	25 (21.9%)
Vitamin B12 deficiency ^c^	n. (%)	11 (9.6%)
Anaemia ^d^	n. (%)	10 (8.8%)
Low serum iron ^e^	n. (%)	23 (20.2%)

^a^ 25(OH)D deficiency values < 20 ng/mL; ^b^ folate deficiency values < 3.0 ng/mL; ^c^ vitamin B12 deficiency values < 200 pg/mL; ^d^ anaemia by haemoglobin values < 12 g/dL (females), <13.5 g/dL (males); ^e^ Low serum iron levels < 50 μg/dL [14], 25(OH)D, 25-hydroxyvitamin D.

**Table 4:** Metabolic differences between individuals with grade-II obesity and those with grade-III obesity.

Parameters	Grade-II Obesity	Grade-III Obesity	*p*-Value
Serum iron levels (μg/dL)	86.38 ± 37.71	72.01 ± 25.74	0.02
Haemoglobin (g/dL)	13.41 ± 3.67	13.75 ± 1.38	0.159
hs-CRP (mg/dL)	0.76 ± 0.53266	1.73 ± 1.75	**0.005**
Fibrinogen (mg/mL)	338.44 ± 72.27	416.37 ± 104.48	**<0.0001**
Glucose (mg/dL)	92.18 ± 19.32	99.31 ± 25.24	0.143
HbA1c (%)	5.52 ± 0.98	5.22 ± 1.69	0.398
Insulin (mIU/mL)	17.07 ± 8.03	23.28 ± 12.75	**0.015**
HOMA-index	3.89 ± 2.14	5.49 ± 2.93	**0.008**
Total cholesterol (mg/dL)	191.09 ± 33.65	191.41 ± 146.7	0.955
LDL cholesterol (mg/dL)	130.34 ± 30.05	134.16 ± 38.7	0.861
Triglycerides (mg/dL)	132.68 ± 70.97	132.06 ± 66.64	0.965
Vitamin B12 (pg/mL)	358.22 ± 130.33	335.56 ± 111.94	0.502
Folic acid (ng/mL)	5.20 ± 3.6	4.01 ± 2.21	0.082
25(OH)D (ng/mL)	19.06 ± 9.75	16.27 ± 9.78	0.193

Data are expressed as mean ± SD. A *p*-value in bold type denotes a significant difference (*p* < 0.05); CRP, c-reactive protein; HbA1c, glycated haemoglobin; HOMA, homeostatic model assessment; LDL, low-density lipoprotein cholesterol; 25(OH)D, 25-hydroxyvitamin D.

**Table 5:** Correlations among serum iron levels, hs-CRP, fibrinogen, BMI, WC, and fat mass %.

Parameters	Serum Iron (μg/dL)	
	r	*p*-Value
hs-CRP(mg/dL)	−0.259	**0.008**
Fibrinogen (mg/mL)	−0.261	**0.006**
BMI (kg/m^2^)	−0.186	**0.04**
WC (cm)	−0.265	**0.004**
Fat mass (%)	−0.285	**0.003**

A *p* value in bold type denotes a significant difference (*p* < 0.05). CRP, c-reactive protein; BMI, body mass index; WC, waist circumference.

**Table 6:** Correlations among hs-CRP, BMI, and WC.

Parameters	hs-CRP (mg/dL)	
	r	*p*-Value
BMI (Kg/m^2^)	0.362	**0.001**
WC (cm)	0.283	**0.004**

A *p* value in bold type denotes a significant difference (*p* < 0.05). BMI, body mass index; WC, waist circumference.

**Table 7:** Multiple regression analysis.

Parameters	Multiple Regression Analysis			
Model 1	R^2^	β	t	*p* Value
**WC (cm)**	−0.772	−0.357	−0.328	**0.001**
**Fat mass (%)**	−1.090	−0.251	−2.272	**0.02**
**hs-CRP (mg/dL)**	−4.974	−0.237	−2.018	**0.04**
Variables excluded: Fibrinogen and BMI				

A *p* value in bold type denotes a significant difference (*p* < 0.05). WC, waist circumference; CRP, c-reactive protein.

## Data Availability

Not applicable.

## Abbreviations

BMI: body mass index; CRP, c-reactive protein; WC, waist circumference; DXA, dual-Energy X-ray Absorptiometry; HbA1c, glycated haemoglobin; LDL, low-density lipoprotein; TG, triglycerides; WHR, World Health Organization; HC, hip circumference; WHR, waist-to-hip ratio; HDL, high-density lipoprotein; HPLC, high-performance liquid chromatography; CLIA, chemiluminescence immunoassay; HOMA, homeostasis model assessment; 25(OH)D, 25-hydroxyvitamin D; IL, interleukin.

## References

[1] WHO (World Health Organization). https://www.who.int/news-room/fact-sheets/detail/obesity-and-overweight.

[2] Cheng H.L., Bryant C., Cook R., O’Connor H., Rooney K., Steinbeck K. (2012). The relationship between obesity and hypoferraemia in adults: A systematic review. Obes. Rev..

[3] Zhao L., Zhang X., Shen Y., Fang X., Wang Y., Wang F. (2015). Obesity and iron deficiency: A quantitative meta-analysis. Obes. Rev..

[4] Kerkadi A., Mohsen Ali R., Shehada A.A.H., Abdelnasser AbouHassanein E., Moawad J., Bawadi H., Shi Z. (2021). Association between central obesity indices and iron status indicators among Qatari adults. PLoS ONE.

[5] Khan A., Khan W.M., Ayub M., Humayun M., Haroon M. (2016). Ferritin Is a Marker of Inflammation rather than Iron Deficiency in Overweight and Obese People. J. Obes..

[6] Flancbaum L., Belsley S., Drake V., Colarusso T., Tayler E. (2006). Preoperative nutritional status of patients undergoing Roux-en-Y gastric bypass for morbid obesity. J. Gastrointest. Surg..

[7] Alshwaiyat N.M., Ahmad A., Wan Hassan W.M.R., Al-Jamal H.A.N. (2021). Association between obesity and iron deficiency (Review). Exp. Ther. Med..

[8] Nuttall F.Q. (2015). Body Mass Index: Obesity, BMI, and Health: A Critical Review. Nutr. Today.

[9] Rosato E., Gigante A., Gasperini M.L., Proietti L., Muscaritoli M. (2021). Assessing Malnutrition in Systemic Sclerosis With Global Leadership Initiative on Malnutrition and European Society of Clinical Nutrition and Metabolism Criteria. JPEN J. Parenter Enteral Nutr..

[10] Muscogiuri G., Barrea L., Laudisio D., Di Somma C., Pugliese G., Salzano C., Colao A., Savastano S. (2019). Somatotropic Axis and Obesity: Is There Any Role for the Mediterranean Diet?. Nutrients.

[11] Barrea L., Muscogiuri G., Laudisio D., Pugliese G., de Alteriis G., Colao A., Savastano S. (2020). Influence of the Mediterranean Diet on 25- Hydroxyvitamin D Levels in Adults. Nutrients.

[12] Matthews D.R., Hosker J.P., Rudenski A.S., Naylor B.A., Treacher D.F., Turner R.C. (1985). Homeostasis model assessment: Insulin resistance and beta-cell function from fasting plasma glucose and insulin concentrations in man. Diabetologia.

[13] Hales C.M., Carroll M.D., Fryar C.D., Ogden C.L. (2020). Prevalence of Obesity and Severe Obesity Among Adults: United States, 2017–2018. NCHS Data Brief.

[14] Pellegrini M., Rahimi F., Boschetti S., Devecchi A., De Francesco A., Mancino M.V., Toppino M., Morino M., Fanni G., Ponzo V. (2021). Pre-operative micronutrient deficiencies in patients with severe obesity candidates for bariatric surgery. J. Endocrinol. Investig..

[15] Vgontzas A.N., Bixler E.O., Papanicolaou D.A., Chrousos G.P. (2000). Chronic systemic inflammation in overweight and obese adults. JAMA.

[16] Fontana L., Eagon J.C., Trujillo M.E., Scherer P.E., Klein S. (2007). Visceral fat adipokine secretion is associated with systemic inflammation in obese humans. Diabetes.

[17] Nemeth E., Rivera S., Gabayan V., Keller C., Taudorf S., Pedersen B.K., Ganz T. (2004). IL-6 mediates hypoferremia of inflammation by inducing the synthesis of the iron regulatory hormone hepcidin. J. Clin. Investig..

[18] Stoffel N.U., El-Mallah C., Herter-Aeberli I., Bissani N., Wehbe N., Obeid O., Zimmermann M.B. (2020). The effect of central obesity on inflammation, hepcidin, and iron metabolism in young women. Int. J. Obes..

[19] Hilton C., Sabaratnam R., Drakesmith H., Karpe F. (2023). Iron, glucose and fat metabolism and obesity: An intertwined relationship. Int. J. Obes..

[20] Iwasaki T., Nakajima A., Yoneda M., Yamada Y., Mukasa K., Fujita K., Fujisawa N., Wada K., Terauchi Y. (2005). Serum ferritin is associated with visceral fat area and subcutaneous fat area. Diabetes Care.

[21] Camaschella C. (2019). Iron deficiency. Blood.

[22] Urbanski G., Chabrun F., Lavigne C., Lacout C., Delattre E., Reynier P., Requin J. (2023). Serum ferritin/C-reactive protein ratio is a simple and effective biomarker for diagnosing iron deficiency in the context of systemic inflammation. QJM.

[23] Wang W., Knovich M.A., Coffman L.G., Torti F.M., Torti S.V. (2010). Serum ferritin: Past, present and future. Biochim. Biophys. Acta.

[24] Dao M.C., Meydani S.N. (2013). Iron biology, immunology, aging, and obesity: Four fields connected by the small peptide hormone hepcidin. Adv. Nutr..

[25] Andrews M., Soto N., Arredondo-Olguin M. (2015). Association between ferritin and hepcidin levels and inflammatory status in patients with type 2 diabetes mellitus and obesity. Nutrition.

[26] Chambers E.C., Heshka S., Gallagher D., Wang J., Pi-Sunyer F.X., Pierson R.N. (2006). Serum iron and body fat distribution in a multiethnic cohort of adults living in New York City. J. Am. Diet. Assoc..

[27] Senkus K.E., Crowe-White K.M., Locher J.L., Ard J.D. (2022). Relative fat mass assessment estimates changes in adiposity among female older adults with obesity after a 12-month exercise and diet intervention. Ann. Med..

[28] Barrea L., Muscogiuri G., Pugliese G., Laudisio D., de Alteriis G., Graziadio C., Colao A., Savastano S. (2021). Phase Angle as an Easy Diagnostic Tool of Meta-Inflammation for the Nutritionist. Nutrients.

[29] Wang W., Feng Y., Long Q., Chen F., Chen Y., Ma M., Mao S. (2022). A comparative analysis of body composition assessment by BIA and DXA in children with type II and III spinal muscular atrophy. Front. Neurol..

[30] Moschonis G., Chrousos G.P., Lionis C., Mougios V., Manios Y., Healthy Growth Study g. (2012). Association of total body and visceral fat mass with iron deficiency in preadolescents: The Healthy Growth Study. Br. J. Nutr..

[31] Kochkodan J., Telem D.A., Ghaferi A.A. (2018). Physiologic and psychological gender differences in bariatric surgery. Surg. Endosc..

